# Assessment of Regional Ventilation Distribution: Comparison of Vibration Response Imaging (VRI) with Electrical Impedance Tomography (EIT)

**DOI:** 10.1371/journal.pone.0086638

**Published:** 2014-01-27

**Authors:** Chang Shi, Stefan Boehme, Alexander H. Bentley, Erik K. Hartmann, Klaus U. Klein, Marc Bodenstein, James E. Baumgardner, Matthias David, Roman Ullrich, Klaus Markstaller

**Affiliations:** 1 Department of Anaesthesiology, Medical Centre of the Johannes Gutenberg-University, Mainz, Germany; 2 Department of Anaesthesia, General Intensive Care and Pain Management, Medical University of Vienna, Vienna, Austria; 3 Beijing Institute of Pharmacology and Toxicology, National Beijing Center for Drug Safety Evaluation and Research, Beijing, China; 4 Oscillogy LLC, Folsom, Pennsylvania, United States of America; D’or Institute of Research and Education, Brazil

## Abstract

**Background:**

Vibration response imaging (VRI) is a bedside technology to monitor ventilation by detecting lung sound vibrations. It is currently unknown whether VRI is able to accurately monitor the local distribution of ventilation within the lungs. We therefore compared VRI to electrical impedance tomography (EIT), an established technique used for the assessment of regional ventilation.

**Methodology/Principal Findings:**

Simultaneous EIT and VRI measurements were performed in the healthy and injured lungs (ALI; induced by saline lavage) at different PEEP levels (0, 5, 10, 15 mbar) in nine piglets. Vibration energy amplitude (VEA) by VRI, and amplitudes of relative impedance changes (rel.ΔZ) by EIT, were evaluated in seven regions of interest (ROIs). To assess the distribution of tidal volume (V_T_) by VRI and EIT, absolute values were normalized to the V_T_ obtained by simultaneous spirometry measurements. Redistribution of ventilation by ALI and PEEP was detected by VRI and EIT. The linear correlation between pooled V_T_ by VEA and rel.ΔZ was R^2^ = 0.96. Bland-Altman analysis showed a bias of −1.07±24.71 ml and limits of agreement of −49.05 to +47.36 ml. Within the different ROIs, correlations of V_T_-distribution by EIT and VRI ranged between R^2^ values of 0.29 and 0.96. ALI and PEEP did not alter the agreement of V_T_ between VRI and EIT.

**Conclusions/Significance:**

Measurements of regional ventilation distribution by VRI are comparable to those obtained by EIT.

## Introduction

Bedside assessment of regional lung function has the potential to optimize mechanical ventilator settings according to individual patient needs. Currently, global parameters such as gas exchange indices and pulmonary compliance are used to assess lung function during mechanical ventilation. Reliable bedside measurement of regional lung dynamics and ventilation distribution, however, could provide additional information about regional heterogeneity [1], [2].

Electrical impedance tomography (EIT) is a technology that has been used in numerous research studies to continuously detect changes in lung ventilation. It is able to measure the regional distribution of tidal volume (V_T_) based on thoracic bioimpedance changes [3]. There is a high linear correlation between EIT and CT in detecting regional ventilation distribution (R^2^ from 0.81 to 0.93) [4], [5]. Furthermore, EIT has been validated against several established methods, such as spirometry (R^2^ 0.98), radionuclide scanning (R^2^ 0.98), and single photon emission CT (R^2^ 0.92) [6], [7], [8]. Despite years of development, EIT has yet to be widely adopted, and alternative methods have been proposed.

Another technology known as vibration response imaging (VRI) has been proposed to dynamically monitor ventilation distribution within the lungs. VRI can be regarded as an “electronic stethoscope”, which records sounds from the chest using acoustic microphones [9] and converts them into grey-scale images [10], [11], [12]. Several studies have demonstrated that VRI technology is an excellent way to detect lung sound distribution during mechanical ventilation in both animal models and patients [13], [14], [15], [16].

VRI has not yet been validated against any established methods, such as CT or EIT, in assessing regional ventilation distribution. We therefore directly compared regional ventilation distribution assessed by VRI with regional ventilation distribution assessed by EIT in animals with normal and injured lungs, and at different levels of positive end-expiratory pressure (PEEP).

## Materials and Methods

### Ethics Statement

This study was carried out in strict accordance with the recommendations in the Guide for the Care and Use of Laboratory Animals of the National Institutes of Health. The protocol was approved by the Animal State Care and Use Committee of the Rhineland Palatinate, Germany (Permit Number: G09-1-029). All surgery was performed under deep anaesthesia, and all efforts were made to minimise suffering [17]. The animal experiments were performed at the Department of Anesthesiology, Medical Center of the Johannes Gutenberg-University, Mainz, Germany.

### Instrumentation

Nine healthy piglets *Sus scrofa domestica* (30±2 kg) were studied. Ketamine (8 mg kg^−1^) and midazolam (0.2 mg kg^−1^) were administered for intramuscular premedication. Anaesthesia was induced using i.v. fentanyl (4 µg kg^−1^) and propofol (2–4 mg kg^−1^). The animals were intubated orotracheally in the supine position using a cuffed endotracheal tube (ID 8.0 mm) facilitated by a single dose of pancuronium (0.15 mg kg^−1^). Ventilation was performed using pressure-controlled mode (PCV). Anaesthesia was maintained by continuous infusion of propofol (6–10 mg kg^−1^ h^−1^) and fentanyl (0.05–0.1 mg h^−1^). Arterial and venous catheters were inserted by surgical cut-down of the femoral artery and vein for haemodynamic monitoring (invasive blood pressures, heart rate) and arterial blood gas analysis (RapidLab 415, Bayer-Healthcare, Leverkusen, Germany). SpO_2_, airway pressures, and inspiratory and expiratory flow curves were dynamically recorded (S/5 Monitoring, Datex-Ohmeda, Duisburg, Germany). Body temperature was kept constant at 38±1°C using body surface warming. The animals were kept in dorsal recumbency for the rest of the experiment.

### EIT and VRI Technology

A 16 electrode EIT system (Goe-MFII study device, Care Fusion, Höchberg, Germany) was used in this study. In order to reproducibly measure a lung section without heart and diaphragm interference, 16 needle-like EIT electrodes were placed in the plane of a transverse cross-section of the thorax, 10 cm cranial to the diaphragm. A reference electrode was placed approximately 4 cm below the acquisition plane. EIT data were generated by applying an electrical current of 5 mA at 50 kHz. Voltage differences between neighbouring electrode-pairs were measured in a rotating sequence at 13 Hz.

**Figure 1:**
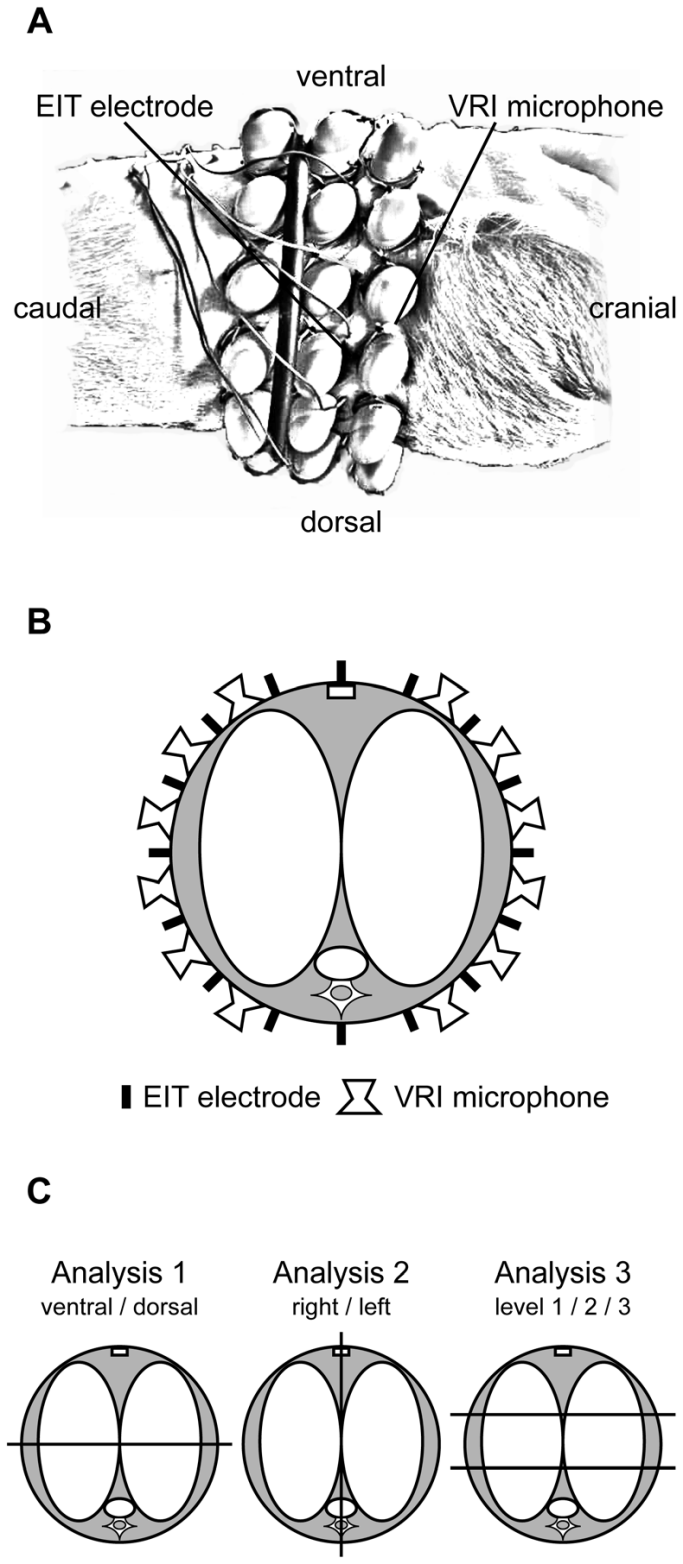
Anatomical sensor position of VRI and EIT. Panel A shows the lateral view of the attached VRI and EIT sensors (derived from photography). Panel B displays schematically the sensor positions in the observed lung cross section. Panel C shows the analyzed ROIs: Therefore, VEA was calculated by the sum of all relevant microphones within the predefined ROIs in accordance to the anatomic position. The three upper rows covered the ventral part, and the three lower rows covered the dorsal part of the lung. The right or left array covered the right or left lung, respectively. For the three transverse ROIs, the two upper microphone rows covered the upper ventral (level 1), the third and fourth rows the middle (level 2), and the fifth and six rows the lower dorsal (level 3) ROIs.

The sensor arrays (six microphones per column by three rows) of the VRI device (VRI_xv_, Deep Breeze Ltd., Or-Akiva, Israel) were placed near the same thoracic area using specialized gel pads. The right and left sensor arrays of the VRI system were laterally placed around the thorax, with the first column of microphones of the VRI sensor arrays cranial, and the second and third column caudal, to the EIT electrodes. The distances between the microphones were predefined by the array matrix. Figure 1 shows the anatomical sensor positions of VRI and EIT.

### Study Protocol

After instrumentation, baseline measurements (BLH) under healthy lung conditions were performed using pressure controlled ventilation (PCV) at a fixed end-inspiratory pressure level (P_endinsp_) of 30 mbar, a PEEP of 0 mbar, an inspiration to expiration ratio (I:E) of 1∶1, and a respiratory rate (RR) of 6 min^−1^. Thereafter, lung injury was induced by repetitive lung lavage with warmed normal saline (30 ml kg^−1^ each) until a PaO_2_ lower than 300 mmHg at a F_I_O_2_ of 1.0 (mild ARDS) and a PEEP of 5 mbar were reached for 1 h. Mean arterial pressure (MAP) was maintained at >50 mmHg with 100 ml boluses of HAES 6%. Impaired lung (ALI) measurements were recorded at different PEEP levels of 0, 5, 10, and 15 mbar in random order, with unchanged ventilator settings. This ventilatory regimen created variation in V_T_ as well as variation in regional ventilation distribution. Each PEEP level was maintained for at least ten minutes before time-synchronised recordings of EIT, VRI, and spirometry were started. Thus, five recordings for each animal (BLH, ALI-0, ALI-5, ALI-10, ALI-15) were performed. After finishing the study protocol, animals were euthanized under deep anaesthesia with a bolus of propofol followed by 40 mmol of potassium chloride.

### Offline Data Handling

**Figure 2:**
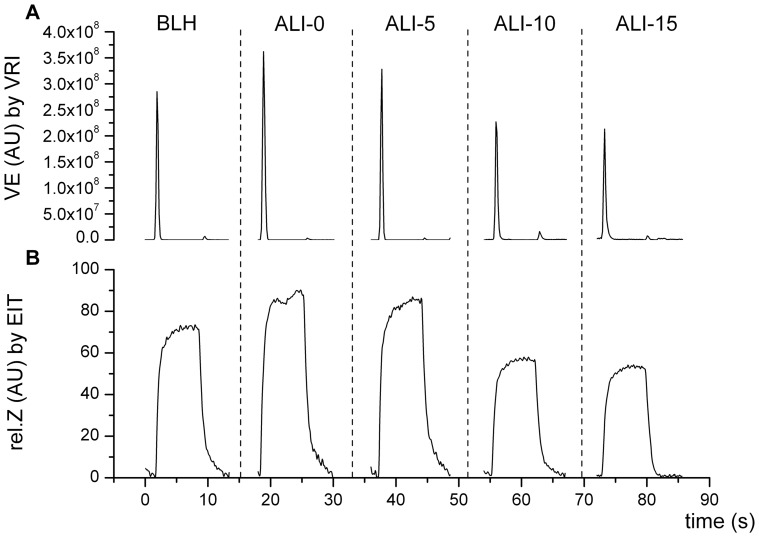
Example of VRI and EIT side-by-side raw data recordings. Raw data waveforms of one representative breathing-cycle for each of the different measurement time points (BLH: baseline healthy; ALI-0: impaired lungs (ALI) at zero PEEP; ALI-5: ALI at PEEP 5 mbar; ALI-10: ALI at PEEP 10 mbar; ALI-15: ALI at PEEP 15 mbar). Panel A: Vibration energy (VE) over time by VRI. The parameter vibration energy amplitude (VEA) was assessed at the peak flow rate of the inspiratory phase of the breathing cycle. Panel B: Relative impedance changes (rel.Z) over time by EIT. The amplitudes of rel.Z (rel.ΔZ) were assessed by tidal differences between minimum and maximum rel.Z values.

For EIT data evaluation, the prototype software SCIEIT (V.1.0, Dept. for Anesthesiological Research, University of Goettingen, Germany) and AUSPEX (V.1.5, Dept. of Physics and Medical Technology, VU Medical Centre Amsterdam, Netherlands) were used. Relative impedance (rel.Z) waveforms were recorded dynamically by EIT (Figure 2B), but were post-processed after the experiments to evaluate V_T_ distribution. EIT waveforms were evaluated by assessing the tidal differences between minimum (end-expiratory) and maximum (end-inspiratory) values. The resulting amplitudes of rel.Z (rel.ΔZ), representing the entire lung cross-section, were evaluated in three different ways: Analysis 1: for ventral and dorsal regions of interest (ROIs); Analysis 2: for right and left ROIs; and Analysis 3: the lung cross-section was equally divided into three parts from ventral to dorsal, resulting in level 1 (upper ventral), level 2 (middle), and level 3 (lower dorsal) ROIs (Figure 1C). To assess regional ventilation distribution, amplitudes of rel.Z were related to total expiratory V_T_ obtained from simultaneous spirometry readings, and calculated for the different ROIs as described by Pulletz *et al.* [18].

The corresponding VRI raw data waveforms were post-processed to evaluate the parameter vibration energy amplitude (VEA) (Figure 2A). This parameter reflects the amplitude of the vibration energy versus the time curve at the point of maximum flow in the inspiratory phase of the breathing cycle. The VRI system derived its vibration energy signals from each of the 34 microphones (2 of the 36 microphones operating as reference) as follows: digitized acoustic signals were band-pass filtered between 150 and 800 Hz to remove heart and muscle sounds; median filtering was applied to suppress impulse noise, which may correspond to artificial sound; and finally the envelope signal was smoothed by running an average filter. The VEA parameter was analysed for the same ROIs used for EIT analysis. VRI ROIs were aligned with EIT as shown in Figure 1. In same manner as described for EIT, we related VEA measured by VRI to the total end-expiratory values of V_T_ obtained from simultaneous spirometry, and calculated regional V_T_ measured by VRI for each ROI.

### Statistical Analysis

Descriptive and statistical data analyses were performed using GraphPad Prism v5 (GraphPad Software Inc, San Diego, CA, USA) and SPSS v18 (IBM Inc., New York, USA). We performed paired measurements of regional ventilation as assessed by VRI, versus regional ventilation as assessed by EIT, in nine piglets. For each data set, there were five paired data points in the analysis corresponding to the five different measurement times (BLH, ALI-0, ALI-5, ALI-10, ALI-15) as described above. Regional ventilation by both methods was compared by linear regression and Bland-Altman analysis [19], [20]. To investigate the impact of lung damage and PEEP on VRI V_T_ measurements, an ANCOVA was performed that tested the equality of slopes and intercepts using an F-test (two-tailed). Thus, a global model where slope is shared among the data sets was compared with a model where each dataset (BLH, ALI-0, ALI-5, ALI-10, ALI-15) gets its own slope [21].

## Results

Overall, regional V_T_ measured by EIT ranged from 57 to 681 ml, while V_T_ measured by VRI ranged from 42 to 688 ml. Additional results of the ventilatory, gas-exchange and hemodynamic parameters are shown in Table 1.

**Table 1:** Ventilatory, gas exchange and hemodynamic parameters in healthy baseline and injured lungs at different PEEP.

	BLH	ALI-0	ALI-5	ALI-10	ALI-15
**P_endinsp_ [mbar]**	24±4	28±3	28±3	29±2	31±3
**PEEP [mbar]**	5±0	0±0	5±1	10±1	15±1
**RR [breaths/min]**	6	6	6	6	6
**V_T_ [ml]**	504±92	686±137	662±131	537±80	437±72
**Crs [ml/cmH_2_O]**	26±4	21±3	23±3	23±3	21±3
**Flow [L/min]**	46±5	53±3	55±4	50±5	50±5
**F_I_O_2_**	1.0	1.0	1.0	1.0	1.0
**P_a_O_2_ [mmHg]**	583±63	269±155	305±132	427±94	447±173
**P_a_CO_2_ [mmHg]**	43±10	47±11	46±11	44±14	49±15
**S_p_O_2_ [%]**	100±0	99±1	99±1	100±0	100±0
**HR [beats/min]**	97±24	95±27	95±25	105±32	112±37
**SAP [mmHg]**	112±21	108±21	113±22	109±23	91±21
**DAP [mmHg]**	70±20	64±15	66±16	66±18	53±10
**MAP [mmHg]**	85±21	80±18	83±20	82±21	66±13
**MPAP [mmHg]**	24±8	32±11	33±10	33±9	32±9
**CVP [mmHg]**	13±5	14±5	15±6	15±6	17±7

N = 9. Values are means ± standard deviations. P_endinsp_: end-inspiratory airway pressure; PEEP: positive end-expiratory pressure; RR: respiratory rate; V_T_: tidal volume; Crs: respiratory system compliance; Flow: airway flow; F_I_O_2_: fraction of inspired oxygen; P_a_O_2_: arterial partial pressure of oxygen; P_a_CO_2_: arterial partial pressure of carbon dioxyde; S_p_O_2_: peripheral saturation; HR: heart rate; SAP, systolic arterial pressure; DAP, diastolic arterial pressure; MAP, mean arterial pressure; MPAP, mean pulmonary arterial pressure; CVP, central venous pressure.

### Changes in Regional Ventilation Distribution Caused by Lung Injury and PEEP

Regional ventilation distribution in right and left, ventral and dorsal, and level 1, level 2, and level 3 lung areas were assessed under varying conditions (healthy lungs at baseline, and injured lungs at different PEEP levels).

**Figure 3:**
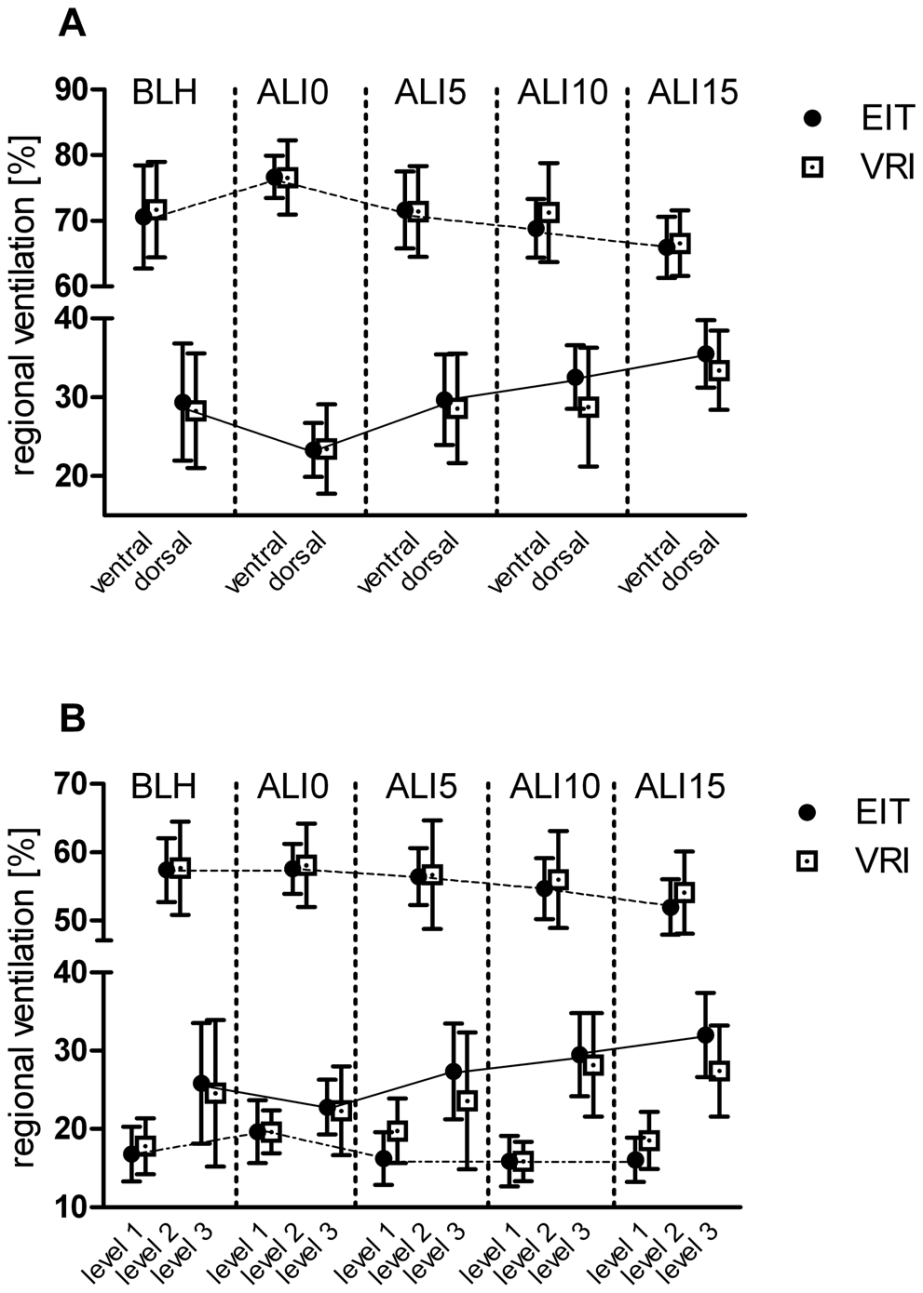
Regional Ventilation Distribution as measured by VRI and EIT. Regional ventilation distribution as measured by VRI and EIT. Values are given as percentage of ventilation as assessed by EIT and VRI, itemized for the different measurement points and respective PEEP levels (BLH: baseline healthy; ALI-0: impaired lungs (ALI) at zero PEEP; ALI-5: ALI at PEEP 5 mbar; ALI-10: ALI at PEEP 10 mbar; ALI-15: ALI at PEEP 15 mbar). Panel A: within ventral and dorsal lung ROI. Panel B: within level 1 (upper ventral), level 2 (middle), and level 3 (lower dorsal) lung ROI.

Total lung volume was almost equally distributed between the right and left lung. Overall, values ranged from 47.5 to 48.8% (EIT) and 47.4 to 50.6% (VRI) for the right lung, and between 51.2 and 52.5% (EIT) and 49.4 and 52.6% (VRI) for the left lung. Induction of lung injury led to a decrease in regional ventilation in the dorsal ROI from 29.4±7.4% (EIT) and 28.3±7.3% (VRI) under healthy lung conditions (BLH), to 23.3±3.4% (EIT) and 23.4±5.6% (VRI) at zero PEEP (ALI-0). Increasing PEEP led to a redistribution of ventilation from ventral to dorsal, which was most pronounced at a PEEP level of 15 mbar (ALI-15); this resulted in a distribution of tidal volume of 35.5±4.3% (EIT) and 33.4±5.0% (VRI) to the dorsal ROI (Figure 3A). No changes in level 1 (upper ventral) ROI V_T_ were observed at different PEEP levels, while increasing PEEP levels shifted regional aeration from level 2 (middle) towards level 3 (lower dorsal) (level 3 ROI: from 22.8±3.5% (EIT) and 22.3±5.7% (VRI) at ALI-0, to 32.0±5.3% (EIT) and 28.2±6.6% (VRI) at ALI-15; Figure 3B).

### Agreement between EIT and VRI

Regional V_T_ distribution for the two methods was highly correlated. In summary, pooled paired measurements of all data sets (Analysis 1; Analysis 2; and Analysis 3) demonstrated that EIT = 12.26+0.96*VRI, R^2^ = 0.96 (*P*<0.0001). Bland-Altman analysis revealed a mean difference between EIT and VRI of −1.07 ml of V_T_ with limits of agreement of −49.05 ml at the lower limit and 47.36 ml at the upper limit.

**Figure 4:**
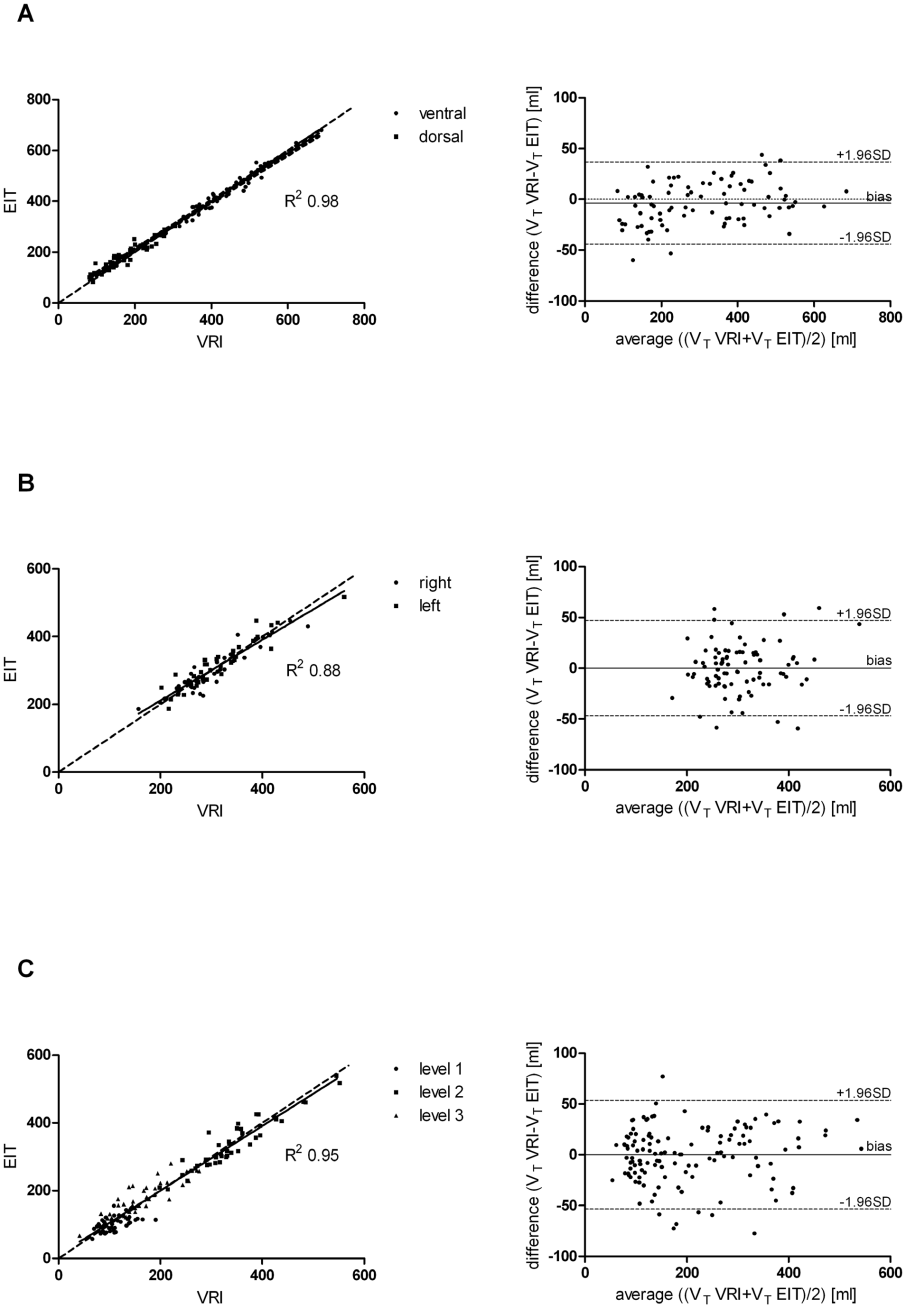
Linear regression plots of estimated V_T_ values by EIT vs. VRI. Linear regression plots of estimated V_T_ values by EIT versus VRI (solid line represents the line of best fit; dashed line shows the identity line) with the corresponding Bland-Altman plots, displaying differences of VRI and EIT V_T_ measurements versus the average (solid line shows the bias; dashed line the limits of agreement). Panel A: Analysis 1 for ventral and dorsal lung regions of interest (ROI). Panel B: Analysis 2 for right and left ROI. Panel C: Analysis 3 for level 1 (upper ventral), level 2 (middle), and level 3 (lower dorsal) ROIs.

Linear regression of Analysis 1 (ventral and dorsal ROI) demonstrated that EIT = 18.84+0.95*VRI, R^2^ = 0.98 (*P*<0.0001). Bland-Altman analysis revealed a mean difference between EIT and VRI of −3.75 ml of V_T_ with limits of agreement of −44.23 ml at the lower limit and 36.74 ml at the upper limit (Figure 4A). Analysis 2 (right and left ROI) demonstrated that EIT = 31.43+0.90*VRI, R^2^ = 0.88 (P<0.0001). Bland-Altman analysis found a mean difference of 0.0 ml of V_T_ with limits of agreement of −44.23 ml at the lower limit and 36.74 ml at the upper limit (Figure 4B). Analysis 3 (upper ventral (level 1), middle (level 2), and lower dorsal (level 3) demonstrated that EIT = 9.64+0.95*VRI, R^2^ = 0.95 (P<0.0001). The corresponding Bland-Altman analysis found a mean difference of 0.0 ml of V_T_ with limits of agreement of −53.58 ml at the lower limit and 53.57 ml at the upper limit (Figure 4C).

Comparing measures of regional ventilation for each of the seven predefined ROIs, there was very good agreement for ventral, dorsal, right, left, level 2 (middle), and level 3 (lower dorsal) lung V_T_-measurements between EIT and VRI (R^2^ from 0.8–0.96) (Table 2). However, agreement in the level 1 (upper ventral) ROI was weak (R^2^ of 0.29).

**Table 2:** Agreement between estimated V_T_ by EIT and VRI for different lung regions: Linear correlation and Bland-Altman analysis.

Lung area	Linear correlation		Bland-Altman analysis	
ROI	Equation	R^2^	Bias±SD	1.96 SD limits: lower/upper
**ventral**	EIT = 5.76+0.98*VRI	0.96	2.32±18.86	−43.56/39.29
**dorsal**	EIT = 36.16+0.85*VRI	0.88	−9.81±20.8	−50.57/30.95
**right**	EIT = 35.39+0.87*VRI	0.88	3.92±24.18	−43.48/51.32
**left**	EIT = 29.92+0.92*VRI	0.89	−3.92±24.18	−51.32/43.48
**level 1**	EIT = 54.68+0.43*VRI	0.29	9.11±24.13	−38.18/56.42
**level 2**	EIT = 24.11+0.92*VRI	0.89	4.59±26.5	−47.36/56.54
**level 3**	EIT = 40.07+0.83*VRI	0.80	−13.7±26.34	−65.35/37.93

Agreement between estimated V_T_ by EIT and VRI for the different lung regions of interest (ROIs): linear correlation equation with the corresponding goodness of fit (left). Bias and 1.96-SD limits of the Bland-Altman analysis (right).

### Influence of Lung Injury and PEEP on EIT and VRI V_T_ Measurement

In order to investigate the impact of ARDS and varying PEEP levels on VRI and EIT assessment of regional V_T_, an ANCOVA was performed. Similar slopes (*P* = 0.07) and intercepts (*P* = 0.85) (Table 3) were obtained for the different data sets (BLH, ALI-0, ALI-5, ALI-10, ALI-15). Thus, lung injury and PEEP *per se* have no relevant impact upon the correlation between EIT and VRI-measured V_T_.

**Table 3:** Influence of lung injury and PEEP on VRI V_T_ measurement: ANCOVA.

		ANCOVA		Differences between VRI and EIT		
Time point	PEEP [mbar]	Slopes [95% CI]	Intercepts [95% CI]	Mean±SD [ml]	Range [ml]	N
BLH	5	0.95 [0.90/1.00]	12.94 [−0.77/26.65]	−0.95±24.39	−77.34/58.41	63
ALI-0	0	0.99 [0.96/1.04]	0.89 [−13.40/15.19]	−0.74±20.95	−59.38/59.30	63
ALI-5	5	0.96 [0.90/1.02]	12.77 [−6.74/32.28]	−1.37±30.91	−72.46/77.10	63
ALI-10	10	0.90 [0.85/0.95]	26.74 [13.46/40.02]	−1.21±24.64	−59.93/44.21	63
ALI-15	15	0.93 [0.87/0.99]	16.31 [1.85/30.78]	−1.04±22.28	−58.59/47.81	63
**Total**	**0 to 15**	**0.96 [0.94/0.98]**	**12.26 [5.95/18.57]**	−**1.07±24.71**	−**77.34/77.10**	**315**
**F-Test (two-tailed)**		***P*** ** = 0.07**	***P*** ** = 0.85**			

Slopes and intercepts for the different measurement points (BLH: baseline healthy; ALI-0: impaired lungs (ALI) at zero PEEP; ALI-5: ALI at PEEP 5 mbar; ALI-10: ALI at PEEP 10 mbar; ALI-15: ALI at PEEP 15 mbar) are presented on the left. Descriptive measures of the differences in V_T_ measured by VRI and EIT, displayed as mean±SD (standard deviation) and range [ml] are presented on the right. To investigate the influence of lung damage and PEEP on EIT and VRI V_T_ measures, an ANCOVA was used to test the equality of slopes and intercepts (using an F test to compare a global model where slope is shared among the data sets, with a model where each dataset gets its own slope). The first *P*-value tested the null hypothesis that the slopes are all identical (the lines are parallel). The second *P*-value represents the results of testing the null hypothesis that the intercepts are identical.

## Discussion

In this study we compared measurements of ventilation distribution by VRI *versus* EIT. Our results show that both methods were highly comparable in their ability to assess changes in regional ventilation distribution (Figure 3). Excellent agreement between VRI and EIT was found for pooled V_T_ measurements (R^2^ 0.96). In the assessment of regional lung ventilation, correlations between the measurements made by VRI and EIT are comparable to studies comparing EIT and CT [4], [5].

In the model used in this study, saline lavage causes pronounced lung collapse due to surfactant depletion, preferentially in dorsal ROI. In addition to the observed decrease in dynamic compliance and airway closure in the injured lungs, de-recruitment can also be attributed to gravity effects in dependent lung regions. This is in agreement with our experimental findings of decreased regional ventilation distribution in dorsal lung areas (Figure 3). With increasing PEEP, the V_T_ redistributed from ventral to dorsal lung areas, as detected by both EIT and VRI. This effect occurred mainly in the middle (level 2) and lower dorsal (level 3) ROIs, reflecting recruitment of atelectasis in dependent lung areas, and consistent with other studies using the saline lavage model [22], [23]. The observed changes in regional distribution were quite small, most likely due to the less severe lung injury (P_a_O_2_/F_I_O_2_<300; mild ARDS) induced by the protocol used in our study. Although we applied relatively high driving pressures during mechanical ventilation, over-distension of non-dependent, upper ventral (level 1) lung areas could not be detected. This finding, however, needs to be carefully interpreted, as we found that there is less agreement at lower V_T_, and therefore over-distension might be underestimated especially at high PEEP levels.

Based on the results by Dellinger *et al.* [14], who demonstrated good correlations between VEA and V_T_ in four healthy human volunteers (R^2^ from 0.74 to 0.82), we presumed that VRI measurements are feasible during PCV. We chose to apply increasing PEEP levels in combination with a fixed end-inspiratory pressure level of 30 mbar in PCV mode to produce varying V_T_ values, and to induce a redistribution of regional ventilation by the same manoeuvre. The various PEEP levels had no relevant impact upon the amplitude of rel.ΔZ, or upon VEA parameters (Table 3).

We chose EIT as the reference method for comparison with VRI, although the accuracy of CT for measuring regional ventilation distribution would have been superior to EIT. We did not, however, use dynamic CT for comparison to VRI since the metallic VRI microphones severely interfere with dynamic CT imaging, making direct and simultaneous comparison impossible. In addition, the VRI device only measures accurately if there is minimal surrounding noise, also precluding the use of CT. Excellent correlations between EIT and CT (R^2^ 0.98) in their ability to track regional ventilation distribution have been demonstrated in previous studies.

This study has several limitations. First, neither VRI nor EIT measure lung volume directly. Based on previous results that showed strong linear correlation of both rel.ΔZ (EIT) and VEA (VRI) with V_T_ [6], [14] for the entire lung, our analysis assumes a linear relationship between regional signal changes from each method, and regional V_T_. Although there is no theoretical bias for this assumption, this simple relationship does allow scaling of the total change in each signal (rel.ΔZ for EIT, VEA for VRI) to an externally measured total V_T_. This empiric assumption has been used before in analysis of EIT data compared to PET [24], which can be considered a reference standard, but has not been applied previously to analysis of VRI data. Thus, the resulting values in ml can only be considered an indirect calculation.

Second, precise alignment of the respective ROIs examined by VRI and EIT was challenging, since the tomograms derived by EIT and the sounds recorded by VRI are not anatomical or spatial measurements. In addition, the low regional resolution (32×32 pixels) of EIT may have biased the results, and could in part explain the observation that differences between VEA and amplitudes of rel.ΔZ are more pronounced at lower V_T_.

There are several methodological limitations with VRI. Sound energy derived from vibrations in large and medium-sized airways is affected by their structural and functional properties, and by the process of being transmitted to the skin after being filtered by lung tissue and the chest wall [25]. Especially in the upper ventral (level 1) lung, VRI sound is affected by turbulence produced at the trachea and carina, which might in part explain the weak correlation between amplitudes of rel.ΔZ and VEA in this ROI.

The pressure controlled ventilation used in the present study resulted in non-constant flow, although our results show only slight differences in mean absolute flow values. It is known that sound energy is affected by both flow through the airways and V_T_ [14], [25]; however, only V_T_ was considered in the analysis of the correlation between VEA and amplitudes of rel.ΔZ. We further limited the analysis to VEA at the peak flow rate of the inspiratory phase. VEA at the peak flow rate of the expiratory phase was simultaneously recorded, but overall expiratory lung sounds have less intensity, resulting in a much lower signal-to-noise ratio with more interference by external noise.

To obtain optimal readings, all measurements were performed under laboratory conditions with minimal surrounding noise. A respiration rate of 6 min^−1^ was used to record more data points within each respiratory cycle. Although the VRI software uses an algorithm to compensate for external noise by automated band-pass filtering of less 150 and higher 800 Hz, it is important to provide similar experimental conditions to obtain optimal readings for clinical use of VRI.

Owing to these methodological limitations of VRI, direct translation of the presented results into clinical practice is limited. Further work will be required to determine the impact of deviations from these controlled conditions on the ability of VRI to assess regional distribution of ventilation.

In summary we conclude that VRI is capable of detecting regional ventilation distribution under pressure-controlled ventilation, under carefully controlled laboratory conditions. The estimated V_T_ by VRI is in excellent agreement with V_T_ as assessed by EIT, with the exception of low V_T_. VRI is capable of detecting changes in regional distribution of ventilation in both healthy and injured lungs, and is not affected by the presence of mild ARDS or varying ventilator settings.
